# Effect of immunoglobulin G concentration in dairy cow colostrum and calf blood serum on *Cryptosporidium* spp. invasion in calves

**DOI:** 10.14202/vetworld.2020.165-169

**Published:** 2020-01-24

**Authors:** Alīna Derbakova, Maksims Zolovs, Dace Keidāne, Žanete Šteingolde

**Affiliations:** 1 Institute of Food and Environmental Hygiene, Faculty of Veterinary Medicine, Latvia University of Life Sciences and Technologies, Kr. Helmana Street 8, Jelgava, Latvia, LV-3004; 2 Department of Biosystematics, Institute of Life Sciences and Technology, Daugavpils University, Parades Street 1a, Daugavpils, Latvia, LV-5401; 3 Animal Disease Diagnostic Laboratory, Institute of Food Safety, Animal Health and Environment “BIOR,” Lejupes Street 3, Riga, Latvia, LV-1076

**Keywords:** calves, *Cryptosporidium*, dairy cows, immunoglobulins

## Abstract

**Aim:**

The research aimed to test the association between the level of immunoglobulin G (IgG) in bovine colostrum and calf blood serum and to evaluate its relation to *Cryptosporidium* spp. invasion in calves.

**Materials and Methods:**

Fresh colostrum and fecal specimens from cows (n=114) as well as blood and fecal specimens from newborn calves (n=114) were collected in the dairy cattle farm. Investigated calves were separated from their mothers directly after birth and received 2 L of colostrum in two separate feedings within the first 24 h. Blood samples were taken from calves at the age of 2 days. Coprological samples were taken from calves at the age of 1, 10, and 15 days. Both colostrum and fecal samples from cows were taken on the 1^st^ day after calf birth. Rectal fecal samples were collected separately from each calf and cow into plastic bags. The collected calf serum samples and bovine colostrum samples were tested for bovine IgG by competitive enzyme-linked immunosorbent assay kit bovine Ig. To record oocysts of *Cryptosporidium* spp. in feces, the flotation method was used. Binomial logistic regression was performed to ascertain the effects of IgG in bovine colostrum and calf blood serum on the likelihood of *Cryptosporidium* spp. infection in calves.

**Results:**

The concentration of IgG in bovine colostrum was higher (70.7±26.6 g/L, mean±standard deviation) than that in calf blood serum (13.2±6.1 g/L); the statistically significant difference was 57.4 g/L (95% confidence interval, 52.4-62.4), t (124.872)=22.536, p<0.001. Mann–Whitney’s U-test showed a significant difference between samples collected on days 10 and 15 of the experiment (U=1944, z=2.330, p=0.020). The higher number of oocysts in calf feces was recorded on day 15 (median=6.5) compared to day 10 (median=4). The prevalence of calf infection from days 10 to 15 increased from 26.3 to 45.6% and was at least 3 times higher than in cows. A statistically significant positive correlation was recorded between IgG concentration of cow colostrum and calf blood serum (r (114)=0.414, p=0.001), whereas a correlation between the concentration of IgG and the intensity of *Cryptosporidium* spp. infection was not recorded (p>0.05). The logistic regression model was not statistically significant (χ^2^(2)=0.013, p=0.99 (10 days) and χ^2^(2)=0.100, p=0.95 (15 days)).

**Conclusion:**

Mother passive transfer of immunity to the offspring through colostrum does not influence the susceptibility of calves to *Cryptosporidium* infestation.

## Introduction

Cryptosporidiosis is a frequent disease in neonatal dairy and beef cattle calves. *Cryptosporidium* spp. cause varying degrees of naturally occurring diarrhea in farm animals. For exaAmple, *Cryptosporidium parvum* is an economically important parasite that causes neonatal diarrhea in calves, lambs, and goat kids. Ignoring the presence of this parasite in farms may result in increased costs for the labor involved in supporting these calves during cryptosporidiosis or even in the death of infected calves [1]. The importance of *Cryptosporidium* spp. is also highlighted by their zoonotic and anthroponotic nature and by the numerous paths of parasite transmission: Water, food, clothes, and footwear. *Cryptosporidium* is a reason for the common cause of acute diarrhea in immunocompetent individuals [2] and recognized as a major waterborne parasite worldwide [3].

The parasites commonly act in concert with other enteropathogens to produce intestinal injury and diarrhea [4]. Colostrum is the first source of newborn food that not only feeds the animal but also provides necessary components for protection. Therefore, receiving adequate colostrum immediately after birth is needed to help prevent the invasion of opportunistic pathogens which can worsen or compound the severity of disease in calves with cryptosporidiosis. Such compounds necessary are immunoglobulins (Ig), which are glycoproteins that specifically recognize and bind to antigens present on pathogens. As Ig have a high degree of specificity, they assist in the destruction of specific pathogens. In animals with the first and second placenta types, such as cows, and Ig are not transferred from maternal blood to the fetus. However, calves receive Ig through passive transfer from colostrum [5]. There are several classes of Ig, including IgA, IgD, IgE, IgG, and IgM [6]; however, most of them are present in colostrum at low concentrations. Of these, IgG are of particular interest because they are the primary Ig found in bovine colostrum and milk and play the main role in the development of humoral immunity [7]. The concentration of IgG in colostrum may reach up to 50–100 mg/ml, and by passive transfer, they provide effective prevention or treatment of several human or animal diseases caused by pathogens (*Yersinia enterocolitica*, *Campylobacter jejuni*, *Escherichia coli*, *Klebsiella pneumoniae*, *Serratia marcescens*, *Salmonella typhimurium*, *Staphylococcus*, *Streptococcus*, *Cryptosporidium*, etc.) [8]. As Ig can prevent the adhesion of pathogens to intestinal epithelial cells, they act as the primal protection against most of the potential gastrointestinal pathogens. For example, IgG by adhesion to invasive stages of *Cryptosporidium* may play a role in preventing invasion into host cells.

The concentration and effect of IgG on calf health have repeatedly been studied [9-14]. For example, Johnsen *et al*. [9] attempted to develop a less invasive and easy method to measure IgG concentration, whereas Aydogdu and Guzelbektes [10] compared colostrum composition between primiparous and multiparous dairy cows. However, only a few investigations of Ig focused on parasitic infections [15,16]. The effects of cryptosporidiosis on calf growth in the long term have not yet been shown, but occurring diarrhea may be costly for farmers due to the loss of income from lower carcass weights. Petry *et al*. [17] noted that the protective role of antibodies is questionable because high titers of parasite-specific IgG can be found in AIDS patients with chronic cryptosporidiosis.

In light of this, this study aimed to test the association between the level of IgG in bovine colostrum and the calf’s blood serum and to evaluate its relation to *Cryptosporidium* spp. invasion in calves. We expected to find an immunological link between cow immunity and the passive transfer of immunity to calves, related to *Cryptosporidium* spp. infection.

## Materials and Methods

### Ethical approval

All procedures performed in studies involving animals were in accordance with the ethical standards. The study was approved by the Animal Welfare and Ethical Council of the Faculty of Veterinary Medicine, Latvia, University of Life Sciences and Technologies, and complied with current laws in Latvia.

### Sample collection

Fresh colostrum and fecal specimens from cows (n=114) as well as blood and fecal specimens from newborn calves (n=114) were collected in the dairy cattle farm between December 2018 and March 2019. Investigated calves were separated from their mothers directly after birth and received 2 L of colostrum in two separate feedings within the first 24 h. Blood samples were taken from 2-day-old calves. Coprological samples were taken from calves at the age of 1, 10, and 15 days. Both colostrum and fecal samples from cows were taken on the 1^st^ day after calf birth. Rectal fecal samples were collected separately from each calf and cow into plastic bags, marked, and kept in a refrigerator at 4°C before examination. If the number of feces was too small (especially in the 1^st^ days of the calves’ life), native smears were made. Before laboratory investigation, blood samples were centrifuged to obtain blood serum and stored at −80°C. The colostrum was stored at the same temperature. During the research, no animals were subjected to unnecessary pain or distress.

### Serological techniques

The collected calf serum samples and bovine colostrum samples were tested for bovine IgG, using the competitive enzyme-linked immunosorbent assay (ELISA) kit bovine Ig (Bio – X Diagnostics, Belgium) in the Institute of Food Safety, Animal Health, and Environment “BIOR,” Serology Division, Latvia. Samples were tested according to the manufacturer’s instructions, and the calibration curve for calf serum and bovine colostrum was established. Calf serum samples were diluted 1/100, and colostrum samples were diluted 1/1000. In the dilution microplate wells, 100 µL of the calibration curve dilutions and diluted samples were transferred, and diluted conjugate was added to each well, mixed, and 100 µL of the content were transferred to the kit’s microplate wells. The microplate was incubated at +21±3°C for 1 h. Subsequently, the microplate was rinsed 3 times with a washing solution, and 100 µL of chromatogen solution were added to each followed by incubation at +21±3°C for 10 min in the dark. The reaction was stopped by adding 50 µL of stop solution to each well. The optical density of the investigated samples was determined using a monochromatic ELISA reader (Thermo Scientific Multiskan FC) with a 450-nm filter. The Ig concentrations were calculated using “Four Parameter Logistic Curve” online data analysis tool, MyAssays Ltd., 10^th^ March 2017, http://www.myassays.com/four-parameter-logistic-curve.assay.

### Coprological examination

All coprological samples were exanimated on the collection day. Laboratory examinations were made in the Laboratory of Parasitology, Institute of Food and Environmental Hygiene, Faculty of Veterinary Medicine, Latvia University of Agriculture. To record oocysts of *Cryptosporidium* spp. in feces, the flotation method was used according to Fujino *et al*. [18]. Slides were stained using the modified Ziehl-Neelsen method [19].

### Statistical analysis

To test the difference between the means of two groups, we used the independent-samples t-test or the Mann–Whitney U-test. The assumption of normality was tested by Shapiro–Wilk’s test and assumption of homogeneity of variances by Levene’s test. To determine the strength and direction of a linear relationship between two continuous variables, we used Pearson’s correlation. Binomial logistic regression was performed to ascertain the effects of IgG in bovine colostrum and calf’s blood serum on the likelihood of *Cryptosporidium* spp. infection in calves. The linearity of the continuous variables with respect to the logit of the dependent variable was assessed through the Box and Tidwell procedure [20]. Bonferroni correction was applied using three terms in the model, resulting in statistical significance being accepted when p<0.016667 [21]. Based on this assessment, all continuous independent variables were found to be linearly related to the logit of the dependent variable.

## Results

A Welch t-test was run to determine if there were differences in the concentrations of IgG between bovine colostrum and calf’s blood serum due to the assumption of homogeneity of variances being violated, as assessed by Levene’s test for equality of variances (F=127.433 p<0.001). Concentrations of IgG for bovine colostrum and calf blood serum were normally distributed (p>0.05). The concentration of IgG in bovine colostrum was higher (70.7±26.6 g/L, mean±standard deviation) than that in calf blood serum (13.2±6.1 g/L); the statistically significant difference was 57.4 g/L (95% confidence interval, 52.4 to 62.4), t (124.872)=22.536, p<0.001. The number of *Cryptosporidium* spp. oocysts in feces was not normally distributed (p<0.05). In the 1^st^ day of the experiment, the prevalence of *Cryptosporidium* spp. was 0%. Mann–Whitney U-test showed a significant difference between samples collected on days 10 and 15 of the experiment; U=1944, z=2.330, p=0.020. The higher number of oocysts in calf feces was recorded on day 15 (median=6.5) compared to day 10 (median=4) of the experiment. The prevalence of calf infection from days 10 to 15 increased from 26.3 to 45.6% and was at least 3 times higher than in cows.

**Figure-1:**
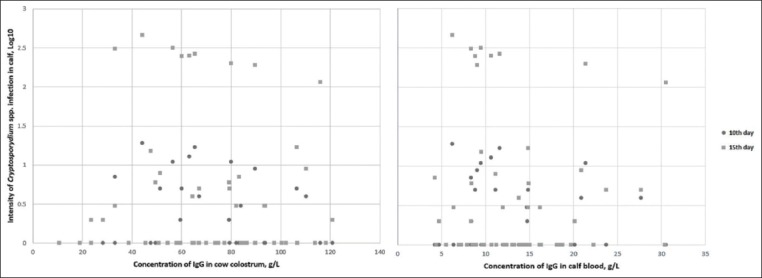
Association between the concentration of immunoglobulin G and intensity of *Cryptosporidium* spp. infection in calves.

A statistically significant positive correlation was recorded between concentrations of IgG in cow colostrum and calf blood serum (r (114)=0.414, p=0.001), whereas a correlation between concentrations of IgG and intensity of *Cryptosporidium* spp. infection was not recorded (p>0.05; Figure-1). The logistic regression model was not statistically significant; χ^2^(2)=0.013, p=0.99 (10 days) and χ^2^(2)=0.100, p=0.95 (15 days), indicating the lack of relationships between the concentration of IgG (in bovine colostrum and calf blood serum) and calf infection with *Cryptosporidium* spp.

## Discussion

The main results showed that calves are born with lower IgG concentrations in the blood compared to cow colostrum. The concentration of IgG in calf blood is directly related to feeding with cow colostrum. Although there is an immunological link between immunity of the mother and passive transfer of immunity to the offspring [22], this study showed the lack of a relationship between concentrations of IgG (both mother and offspring) and *Cryptosporidium* spp. infection. Surprisingly, there was an increase in the infection prevalence of *Cryptosporidium* spp. because colostrum and milk contain not only IgG but also a range of other components such as neutrophils, macrophages, lymphocytes, antimicrobial factors, and other molecules that provide energy for an effective immune response [23-25].

The immune responses to *Cryptosporidium* species infection involve both innate and adaptive immunity. Numerous studies have described the role of T and B cells, intestinal epithelial cells, interferon-gamma and natural killer cells, nitric oxide, antimicrobial peptides, prostaglandins, mannose-binding lectin, cytokines, chemokines, dendritic cells, and macrophages in the formation of immune responses to *Cryptosporidium* spp.; a summary of these studies may be found in the reviews of Leitcha and Heb [26] and Vanathy *et al*. [27]. Our study indicates that innate and adaptive immunity play more significant roles in immune responses to *Cryptosporidium* species than mother passive transfers of immunity to the offspring. Furthermore, Siachos *et al*. [28] suggest that passive transfer of Ig is not protective against cryptosporidiosis. However, Ajjampur *et al*. [29] have studied antibody responses to specific antigens (gp40 and gp15) before and after the first episode of symptomatic cryptosporidiosis in children and found significant increases of IgG levels in response to *Cryptosporidium* antigens. Similar results have been obtained by Allison *et al*. [30], who studied antibody responses to the immunodominant gp15 antigen from *Cryptosporidium hominis* and *C. parvum*. They also recorded that IgM response occurs immediately after an acute infection and levels decrease within weeks, whereas IgG responses are slower to appear but persist for a longer period.

Previous studies of *Cryptosporidium* prevalence in the USA [31] and China [32] coincide with our findings that calves have a higher prevalence of infection than cows. For example, Santín *et al*. [33] concluded that the prevalence of *Cryptosporidium* species is age-related between pre-weaned and post-weaned calves. Harp *et al*. [34] demonstrated that initial exposure of *C. parvum* to calves (from birth to 3 months) and their recovery renders calves resistant to further challenge with the parasite. Most likely, these findings indirectly highlight the significance of adaptive immunity to *Cryptosporidium* infection. However, Gong *et al*. [32] have indicated that *Cryptosporidium* species/subtypes vary among the different age groups of cattle, suggesting that infection with a single species of *Cryptosporidium* and further recovery do not guarantee the inability of cryptosporidiosis caused by other species. Thomson *et al*. [35] have suggested that the ability of the parasite to infect the gut is linked to changes in the gut microflora during animal maturation, although there are no experimental trials demonstrating this in cattle.

## Conclusion

We found that mother passive transfer of immunity to the offspring through colostrum does not influence the susceptibility of calves to *Cryptosporidium* infestation.

## Authors’ Contributions

AD developed the study design and carried out the experiment. ZS performed calf serum and bovine colostrum sample serological examination. DK supervised the research process. MZ performed the statistical analysis of all data. AD and MZ wrote the manuscript with input from all authors. All authors read and approved the final manuscript.
